# Surveillance Review System to Track Progress Towards Polio Eradication in the Horn of Africa

**DOI:** 10.29245/2578-3009/2021/S2.1111

**Published:** 2021-04-16

**Authors:** Samuel Okiror, John Ogange, Hemant Shukla, Christine Lamoureau, Mwaka Monze, Amina Ismail, Anthony Kazoka, Ben Nkowane, Raoul Kamadjeu, Obianuju Igweonu, Joseph Okeibunor, Chidiadi Nwogu

**Affiliations:** 1 WHO Horn of Africa Coordination Office (HOA), Nairobi, Kenya; 2 World Health Organization, Kenya Country Office; 3 WHO Headquarters, Geneva; 4 Independent Consultant; 5 Polio Laboratory, Zambia; 6 World Health Organization, Tanzania Country Office; 7 UNICEF, New York; 8 University of Nigeria, Nsukka; 9 WHO Regional Office for Africa (WHO AFRO) Brazzaville, Congo

**Keywords:** Surveillance review, Acute flaccid paralysis, Poliovirus, Reviews, Horn of Africa

## Abstract

**Background:**

The risk for importation and reintroduction wild poliovirus in areas that have been cleared of the wild poliovirus in the Horn of Africa will remain if the surveillance systems are weak and porous.

**Methods:**

Consequently, the Horn of Africa Polio Coordinating Office in Nairobi, together with partners conducted surveillance reviews for some of the countries in the Horn of Africa, especially Ethiopia, Kenya and Somalia to identify gaps in the polio surveillance and provided recommendations for improved surveillance. Structured questionnaires collected information about acute flaccid paralysis (AFP) surveillance resources, training, data monitoring, and supervision at provincial, district, and health facility levels. Other information collected included resource availability, management and monitoring of AFP surveillance.

**Results:**

The result revealed that although AFP surveillance systems were well established in these countries, a number of gaps and constraints existed. Widespread deficiencies and inefficient resource flow systems were observed and reported at all levels. There were also deficiencies related to provider knowledge, funding, training, and supervision, and were particularly evident at the health facility level. These weaknesses were corroborated with the sustained transmission of polioviruses in the region, where the surveillance systems were not sensitive enough to pick the viruses.

**Conclusion:**

The review teams made useful recommendations that led to strengthening of the surveillance systems in these countries, including the formation and use of village polio volunteers in the south and central zones of Somalia, where security was heavily compromised and surveillance officers lacked regular access to the communities.

## Introduction

The World Health Assembly of the World Health Organization (WHO), in 2012, declared completion of polio eradication a programmatic and public health emergency^1–3^. The Global Polio Eradication Initiative (GPEI) was set up with the mandate of completing the eradication and containment of all polioviruses, such that no child ever again suffers paralytic poliomyelitis^4^. Surveillance of acute flaccid paralysis (AFP) cases was the primary process for detecting polio cases. When detected, stool specimens are tested for polioviruses (PVs) at WHO-accredited laboratories within the Global Polio Laboratory Network (GPLN). Environmental surveillance supplements AFP surveillance and it entails testing sewage samples from selected sites for PVs. Here, virologic surveillance, including genomic sequencing to identify isolates by genotype and measure divergence between isolates, guides Global Polio Eradication Initiative (GPEI) activities by confirming the presence of PV, tracking chains of PV transmission, and highlighting gaps in AFP surveillance quality^3^.

Between 2013 and 2014, there were devastating outbreaks of polioviruses in the Horn of Africa. It started in April 2013, with detection of a case in Banadir region of Somalia. The outbreak rapidly spread to other district in Somalia and to bordering areas of Kenya and Ethiopia^5,6^. The last case of the WPV outbreak was detected in Puntland Somalia in August 2014. A total of 223 cases were confirmed (10 in Ethiopia, 14 in Kenya and 199 in Somalia). The countries in the Horn of Africa have experienced previous outbreaks due to importation of WPV1. The most recent outbreaks prior to the current one was from 2004 – 2008 and 2009 - 2012. The 2013-2014 outbreaks occurred in a complex emergency setting with spread facilitated by inaccessibility, insecurity and massive population movement. This outbreak was therefore indicative of the weaknesses in the surveillance system.

A declining AFP surveillance core indicators performance was for instance observed in Kenya since 2013. The 14^th^ and 15^th^ Horn of Africa Technical Advisory Group meetings recommended that, an external surveillance review be carried out since the last external surveillance review was conducted almost 5 years ago (February 2012) before the devolution of the government system. The last indigenous Wild Polio Virus (WPV) was detected in Kenya in 1984. However, the country experience 4 importations of WPV outbreaks in 2006, 2009, 2011 and 2013. The most recent WPV outbreak in 2013 was detected in Dadaab Refugees camp and host communities where 14 cases were confirmed with WPV1 of which 3 were adults. The date of onset of the last case was on 14th July, 2013 and from that outbreak Kenya conducted immunization response by implementing 15 rounds of SIAs (using tOPV, bOPV and IPV) from 2013 to 2015, one was to all age groups in selected Counties. This was a typical situation across the countries in the Horn of Africa.

Understandably, therefore, with the creation of the Horn of Africa Coordination office, surveillance reviews were some of the actions prioritized to ensure efficient surveillance for AFP. The reviews were designed to assess the organization and the implementation of disease surveillance at all levels in the light of the devolved system of governance with a particular focus on vaccine-preventable diseases (VPDs). Attention was also to be given to the sensitivity and functionality of AFP surveillance at all levels. The above was to be conducted with an aim to determine the strengths, weaknesses, threats and opportunities in the disease surveillance system in addition to pinpointing factors responsible for the sub-optimal performance of AFP surveillance, especially at the Sub-County level to be able to provide recommendations to all stakeholders to address the observed gaps.

This paper provides AFP surveillance quality indicators at national and subnational levels during 2012–2013 for countries that experienced PV cases during 2013-2014 in the Horn of Africa, the remaining polio-endemic region^7^. To achieve polio eradication and certify interruption of PV transmission, intensive efforts to strengthen and maintain AFP surveillance are needed at subnational levels, including in field investigation and prompt collection of specimens, particularly in countries with current or recent active PV transmission.

## Methods

The selection of Counties and Sub-Counties was made based on AFP risk analysis of high, medium and low risk factors, and some based on security conditions, which allowed safe traveling. Health facilities within Sub-Counties were selected purposively. The WHO tools were adapted after consultations with ministry of health. Data collection method included interviews with key informants and document reviews. The national and sub national levels were also reviewed in Ethiopia and Somalia using the same approach as described for Kenya above.

## Results

### Summary of AFP surveillance performances

**Figure 1:**
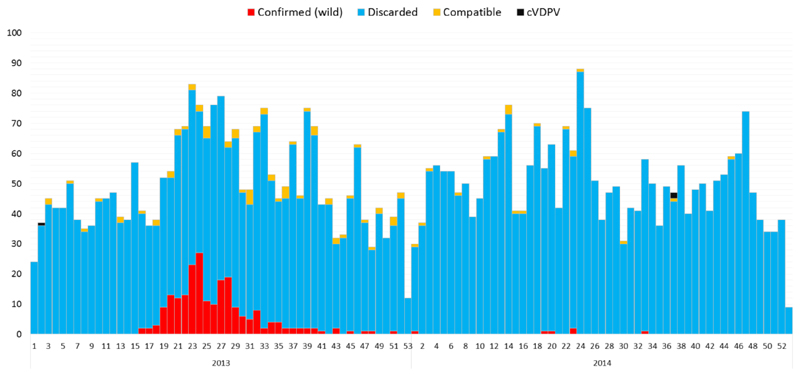
Reported AFP cases in the Outbreak Countries by Classification 2013-2014

**Figure 2:**
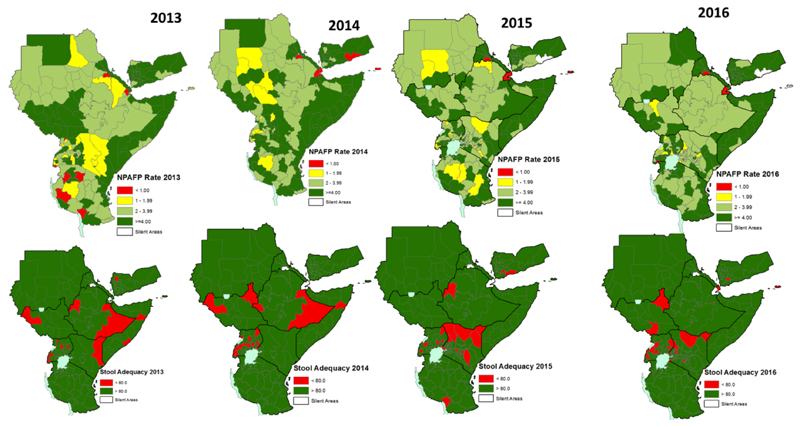
Reported AFP cases in the Outbreak Countries by Classification 2013-2014

**Figure 3:**
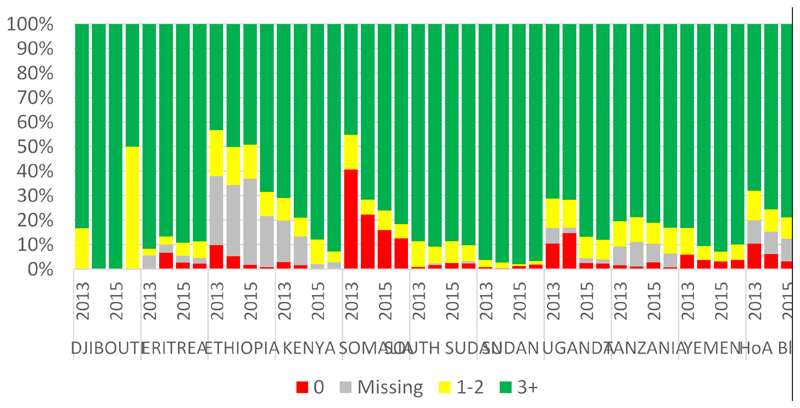
Immunity Profile NP- AFP cases, 6-59 months, 2013 -2016

The WPV and cVDPV has not been reported in Kenya in the one year surveillance review period. However, during the period some of the AFP cases were delayed (pending) to be finally classified since week 3 (Figure 1). There is no silent county and most of the county reported NP-AFP rate more than 2/100,000 population aged less than 15 years. However, almost one third of counties have a stool adequacy of less than 80%. In spite of the sub-national level performances of the two core AFP surveillance indicators, there were also silent and sub-optimal performances at the lower levels (Figure 2). Furthermore, there were also reported AFP cases with missing or no vaccination status (Figure 3).

### Surveillance structure and organization

The Surveillance system structure was well established from the National level down to the community level. NPEC, NCC and NTF committees are functional and there are designated surveillance focal points at all levels from the National to the Sub-County level. However, only 89% of the Health facilities visited have designated surveillance Focal Points. Written terms of reference were available at County (80%) and Sub-County level (55%). In addition, written surveillance work-plans were seen at County level (90%) and Sub-County level (70%). Operational guidelines for AFP, Measles, VPDs and IDSR were available in all Counties and in 95% of Sub-Counties. Availability of reference documents at County level was 100% and at the Sub-County level was 70%. Surveillance activities were integrated with other public health activities, e.g. Malaria, Maternal health services, etc. in some Counties and Sub-Counties.

The National surveillance action plan was not activity-specific; timelines and deliverables were absent as were specific budget lines for surveillance. However, budget lines were available at only 40% of Counties and 25% of Sub-Counties. Sixty-day follow-up reports were missing for most inadequate AFP cases (14 reports conducted /60 inadequate cases), and surveillance review meetings were not held regularly (40% of counties and 70% in sub counties).

### Strengths of the surveillance systems in the Horn of Africa countries

Some the strengths of the surveillance system in ***Kenya*** include the fact that the surveillance system structure was well established from the National level down to the community level. NPEC, NCC and NTF committees are functional and there are designated surveillance focal points at all levels from the National to the Sub-County level. However, only 89% of the Health facilities visited have designated surveillance Focal Points. Written terms of reference were available at County (80%) and Sub-County level (55%). In addition, written surveillance work-plans were seen at County level (90%) and Sub-County level (70%). Operational guidelines for AFP, Measles, VPDs and IDSR were available in all Counties and in 95% of Sub-Counties. Availability of reference documents at County level was 100% and at the Sub-County level was 70%. Surveillance activities were integrated with other public health activities, e.g. Malaria, Maternal health services, etc. in some Counties and Sub-Counties.

Prioritization of surveillance reporting sites was available (high, medium and low) at County (90%) and Sub-County level (85%). Monitoring of the completion and promptness of reporting was done electronically through eIDSR, which recently was migrated to DHIS. Moreover, IDSR tools were available in all Counties and 95% of the visited Sub-Counties. However, only 80% of visited Counties and Sub-Counties submitted the weekly IDSR reports to National level. Majority (57%) of Health facilities visited had awareness creation programs, and a review of the records in the facilities showed no missed AFP or measles cases. Health workers were knowledgeable about case definitions for AFP (83%), measles (88%), NNT (66%) and YF (47%). Surveillance posters on case definition for priority diseases were found in most Sub-Counties and Health facilities.

There was an integrated checklist for supportive supervision, and 75% of Health facilities received supervisory visits with feedback given in 54% of the same. There was a marked reduction in the proportion of AFP cases with unknown immunization status from 17% in 2013 to 3% in 2016. Surveillance focal points possessed good knowledge for stool collection procedure in Counties and Sub Counties (100%) as well as in Health facilities (66%). The polio and IDSR updates were prepared weekly at the National level. In addition, at National, County (90%) and Sub-County level (90%) AFP and measles case-based data were well maintained with tools. Furthermore, 80% of Counties and 65% of Sub-Counties had a tool for recording outbreaks and/or rumours. Stool specimens were shipped from the field to national level within the recommended timelines with 88% of samples reaching the lab within 72 hours after the second stool collection. Specimen collection stool kits were obtainable at the Sub-County level. Weekly reporting forms were available in 92% of Health facilities.

There was a presence of a highly committed technical team. Surveillance was perceived as a priority health program by 90% of the Counties and 75% of health workers. Community Health volunteers were linked to all Health facilities. In addition, 82% of Health facility surveillance Focal Points responded that the community health volunteers were expected to report priority diseases furthermore, the community based surveillance activities are planned in 60% of the visited health facilities. It was observed that regular harmonization meetings were conducted between the laboratory and the surveillance program. Written SOPs on data flow and managements were available and data were widely obtainable at County and Sub-County level in electronic formats. 70% of the Counties visited had written procedures for handling of surveillance data.

Focal Points knew that AEFI was under surveillance, County (80%), Sub-County (85%) and Health facility (81%). Weekly reporting was conducted through IDSR. The documentation provided by the secretariat (MOH & WHO) for NPEC meetings is inadequate. The following were among major deficiencies observed; Sixty days follow up reports are not provided, results of contact sampling are not taken into account, there is no consideration given to hot cases, clinical records are not consulted, and clinical diagnosis is not provided. Thirty six inadequate AFP cases were yet to be classified by NPEC (some cases dating back to January). Only 2 NPEC meetings have been held in the past 12 months.

In ***Ethiopia***, the review team noted that there is a well-established Surveillance structure in all regions with designated Public health emergency management (PHEM) focal persons at all levels. There were availability of PHEM guidelines at regional and Woreda levels, and most of the health facilities visited. Training of PHEM focal persons on surveillance has been undertaken within the last 12 months in most of the regions and availability of Surveillance work plans at the Regions and Woreda levels. Surveillance is considered as a priority health activity in all the regions. High level of awareness about case definitions of AFP, Measles & Neonatal Tetanus and the national procedure for investigations, Posters with case definitions seen in all facilities visited and completeness of weekly reports was generally above 80%. In the overall, Ethiopia was adjudged as having high sensitivity of AFP and measles surveillance in the reporting network. The health facility surveillance focal officers do the job before sending weekly report to the next level. Guidelines on active surveillance visits as per prioritization, supervisory plans and integrated checklist were found to be available at most of the places visited.

Overall quality of case investigation was good; above 95% of the 80% of all AFP cases validated had no major mismatch improved timely notification, stool collection and transport to laboratory. Weekly bulletin were produced and shared with stakeholder in all regions with the exception of Addis Ababa, availability of Weekly reports in nearly all health facilities, Woredas and Zones visited, CIF available in the zones, woreda and HF level, archiving and filing of CIFs, supervisory, weekly and feedback reports at Zonal/ Woreda level were good in Addis, Gambela, Oromia, Tigray, BGZ, and SNNP regions and documentation of timeliness of reports were observed at the Woreda level. The team noted a very good tracking mechanism for temperature and AFP stool samples conditions, high knowledge of shipment procedures, availability and prepositioning of kits at all levels except in Gambela where it was only at the region level where it cannot be easily transported to the lower levels at the time of needs.

The team observed a functioning integrated surveillance system at all levels with the availability of NNT/Measles guidelines, forms and Kits prepositioned at various levels and a high knowledge of standard case definitions for NNT and Measles for case detection among staff. Ethiopia has exhibited excellent ownership of surveillance programs and reinforced with commitment at all levels with PHEM taken up active roles at all levels. The introduction of Health extension workers and health development army into the scheme of community based surveillance in most regions, though started recently in Somali region with 2 Volunteers/ Kibele, has helped surveillance at the community level. The regular meetings between the health extension workers and the health development armies in most regions has also improved exchange of surveillance information, channeling of activity in the right direction with a good outcome. 12 NGOs were found to be working with 14,423 Community Volunteers and health development Armies in 86 Woredas of 5 regions serving a population of 5.7 million. Monitoring of timeliness and completeness of weekly reports were universally done in all regions with regular good quality data analysis at the National levels. Bulletin were also produced and shared regularly.

The EPI focal persons were aware of AEFIs. Three committees of the NCC, NTF and NPEC are in place and their chairpersons trained in 2014. High standard public health laboratory support for vaccine preventable diseases surveillance was found in place and have for many years been accredited by WHO for AFP and measles with good attention to quality assurance processes. The Measles laboratory has been decentralized with two sub-national laboratories fully operational. The operational arm had recently undergone renovations and some other newly constructed laboratory infrastructure in place. Laboratory support for new vaccine surveillance has been established and referral of isolates for genotypic tracking commenced.

Some of the strengths of the surveillance system in *Somalia* include the regular review of the guidelines at central level in Nairobi. This however needs further development to ensure consistency with global and regional guidelines. The training material that was distributed in Mogadishu on the District Polio Officers (DPOs) in December 2012 was available at peripheral level. Members of staff at all levels are dedicated and committed despite all the challenges. There is stable staffing and all positions are filled with clear roles and responsibilities. DPOs as well as Polio Eradication officers (PEOs) have good contact with the community and understand their roles and responsibility. Investigation forms were seen in the regional office and with the regional PEOs who also have copies of the monthly zero reporting.

Prioritization of sites is not well defined and in Sahil and Banadir accessibility directs selection of sites. Surveillance sites are continuously reviewed and list is updated every 6 months. Staff is reasonably qualified and knowledgeable for their jobs. Generally, good level of awareness among medical staff, para-meds, and management. Good awareness of the para-medical staff examining < 5years children in Banadir maternal child health (MCH) facilities. All visited facilities had a focal point/contact person identified for AFP surveillance. Case definition was known and available. Staff members knew who to report to in MCH facilities. PEOs have good rapport with health workers in all visited facilities. MCH records include a limited number of simple diagnoses as they are run most of the time by parameds and sometimes by non-medical staff with limited or no medical background (Banadir). Supervisory visits are mainly from zone to regional staff and from regional to DPOs. Sensitization of communities is continuous through the Village Polio Volunteers who are locally based and through the radios that are communally listened to.

Files of all AFP case were available and complete at zonal/region level. DPOs are notified by Village Polio Volunteers when an AFP Case is detected and investigation is usually done on time within 48 hours by PEOs. In certain accessible areas a clinician participates in the case investigation. AFP performance Indicators are maintained at national level. Central level updates and bulletins are prepared and shared with Partners. Surveillance case line list is shared up to Regional level with regional PEOs. Strong communication between all levels using mobile phones and email as one of mechanism of feedback. There is regular contact between zone PEOs and central level in Nairobi and between Zonal PEOs and PEOs in regions mainly through e-mail and Teleconferences. Mapping of accessible and inaccessible areas is conducted and disseminated for use. Zero reporting sheets were available by week for the different facilities.

There is demonstrated high level support for the AFP Surveillance system by the Ministry of Health as well as the Governors. At both these levels the highest officers are always available to discuss the achievements as well as the challenges and depending on the circumstances, they provide the necessary support. The surveillance system in Somalia is based on the arrangement of the village Polio Volunteers who are community based and detect nearly 70% of all AFP cases. Data on surveillance is collected at all levels and managed mainly at the National Level and with Partners. There is no system of AEFI set up in Somalia as the only functional surveillance system is the one set up for AFP surveillance and also picks up other vaccine Preventable Disease occurrences.

### Weaknesses in the surveillance systems in the Horn of Africa countries

Some of the challenges noticed in the ***Kenyan*** surveillance system include the realization that the national surveillance action plan was not activity-specific; timelines and deliverables were absent as were specific budget lines for surveillance. However, budget lines were available at only 40% of Counties and 25% of SubCounties. Sixty-day follow-up reports were missing for most inadequate AFP cases (14 reports conducted /60 inadequate cases), and surveillance review meetings were not held regularly (40% of counties and 70% in sub counties). Only 26% of Community Health Volunteers (CHVs) were regularly reporting weekly surveillance activities. In addition, there is inadequate involvement of private health facilities in some of the counties as well as traditional healers and alternative therapy. It was also observed that integrated case investigation forms (MOH502) were available in only 60% of the Health facilities and the new AFP Case Investigation form (CIF) was not in use in the whole Country.

Only 50% of Sub-County surveillance Focal Points were trained on IDSR. In the health facilities visited; It was also realized that a varying percentage of the focal points were unaware of the procedures for investigation, sample collection, sample referral and response for AFP (34%), Measles (36%) and Yellow Fever (69%). In addition, 68% of clinicians were inadequately sensitized or have not been trained on surveillance in the last 12 months. There was no tool to document community or clinician sensitization. Active surveillance was not conducted as per the guidelines. An integrated supervision checklist was available in only 40% of the Counties and 30% of the Sub-Counties. Supervisory visits were not conducted as per plans, mainly due to resource constraints. The supervisory checklist includes surveillance of only 40% of the Counties and 50% of the Sub-Counties. Moreover, 40 out of 47 counties have not conducted IDSR training in the past one year.

There was an absence of a national protocol for contact sampling, and the AFP contact sampling was very irregular. Of the 519 AFP cases in 2016, only 45 contact samples were collected. Twenty-two percent of the AFP cases were investigated beyond the required timeline after notification (24-48H), and two cases have been investigated after a period of two months since the date of notification. Validation of AFP cases were not initiated since the 15^th^ HOA TAG recommendation. Case investigation and stool collection processes often wait for the sub-county surveillance focal persons to visit the cases.

There was a general lack of analysis and feedback of data on operational surveillance aspects, at County (80%) and Sub-County (80%) levels. Case-based surveillance forms were not updated with the laboratory results and EPID Number at County, Sub-county and Health facility levels. The stool specimens and case investigation forms (CIF) were sent directly to the laboratory and program receives the CIF after at least a week. During this period, the national surveillance program was unaware of any AFP/measles cases identified and was therefore, unable to take any prompt action. Access to available transport and funding for fuel at County and Sub-County level for surveillance activities remained a challenge. Reimbursement of funds used for specimen shipment from the field was often delayed and, in some cases, simply not paid. Funds provided by WHO for surveillance, given to the MOH, were delayed in reaching the operational level. The funds advanced by WHO in 2015 were yet to be accounted for.

The advocacy for disease surveillance to the county assembly is inadequate. 60% of counties and 75% of Sub-Counties had no budget allocations for disease surveillance and staffs were getting demotivated due to general lack of support. Surveillance was not well integrated with the existing community health volunteers who were focused more on other donors supported programs. There was no sensitization on surveillance during the various monthly community health volunteers’ meetings. In most facilities, there were no regular meetings with community health volunteers, and most traditional healers as well as alternative therapists were not linked to surveillance activities.

There is a lack of updated line graph for VDP found only in 30% of the visited counties, 5% of sub counties and 18% of visited health facilities. There is no evidence of basic analysis for case-based surveillance in all of the visited counties and sub counties. There were discrepancies between the DHIS and AFP surveillance database. Nine out of ten reported AFP clusters notified in 2016 were not investigated as shown in Figure 6. There was a very low proportion (20%) of written procedures for handling of surveillance data at Sub-County level. Guidelines for AEFI were not available in 60% of the Counties, 65% of Sub Counties and Health facilities. No AEFIs have been reported at sites visited.

In ***Ethiopia***, the observed weaknesses include the fact that in all regions visited by the team, there was high Staff turnover, lack of adequate resources for active surveillance and Surveillance focal persons at Woreda level having other additional responsibilities. Irregular review and monitoring meetings were held in many regions to establish surveillance outcomes and actions. Lack of functional Zonal structure felt in Somali and Gambella region, and Woreda structure in Addis Ababa. There was lack of systematic reporting protocol from refugee camps to Regional Health Bureaus and some of focal persons at Woreda level still not trained. There were poor documentation practices with regards to review of prioritization of network sites at the lower levels at the regions. Most Traditional healers/ Holy water sites are not part of the network in Addis and some areas of Oromia and SNNPR regions including relevant private facility practitioners not included in reporting network in Somali and SNNPR regions. Some relevant private practitioners were omitted in the reporting network in Somali and SNNP Regions. The Knowledge gap in calculation of the surveillance core indicators in most of the regions, poor knowledge level on Yellow fever and Adverse Events Following Immunization (AEFI) surveillance was clearly evident. There were also palpable evidence of lack of clarity on diseases under surveillance among some of clinicians in some of the health facilities. There are still possibilities of missing AFP cases as evidenced by detection of unreported AFP cases in 3 regions of Somali, Tigray and Gondar.

Active surveillance visits not being conducted as per plan or national guidelines, Quality of active surveillance visits were found to be suboptimal in the areas of feedback, capacity building and reviewing of appropriate registers in all relevant units in all the regions visited. The mechanism for tracking/ monitoring active surveillance visits by Woreda is not uniform except in Benishangul Gumuz Region. Supervision visits to the next lower level is inadequate and even when/where there is it lacks documentation that such visits took place however at the Woreda level and health centre levels of SNNPR visits were documented. Sensitization of Clinicians on reportable diseases was not regular. Only 50% of index AFP cases had their contact samples taken, the program is plagued with High percentages (36%) of unknown doses on first investigation which was reduced to 8% on validation. Time lag between investigation and validation of AFP cases is longer than normal and remains a concern to the program.

Observations were made of the absence of hard copies of weekly reports in some health facilities and Health posts in Somali and Oromia regions, laboratory feedback, EPID number and findings from validation in CIF from the field were not documented. PHEM do not have copies of CIFs to cross check the line list. Lack of Transport at all levels limited movement of both human and material resources for active surveillance visits. For Yellow fever, there were no guidelines and poor knowledge of standard case definition and procedures for sample collection exists. The team observed poor knowledge of calculation of core surveillance indicators, analysis and information not used for actions. There was also inadequate awareness of appropriate response when NNT case is identified in Addis, North Western Tigray, Benishangul Gumuz, Oromia and Gambela Regions. Furthermore, there has been inadequate resource allocation for surveillance activities which the team observed as a big concern to the smooth running of the program.

There is no structural involvement of PHEM in Community Based Surveillance, health development Army structures do not exist in all places, like in Somali region the epi-centre of outbreaks in Ethiopia and measurement of impact of Community Based Surveillance through recording source of cases reported by the HDAs has not been standardized. The absence of PHEM Data Manager at National level continue to affect program with key surveillance indicators not analyzed and seldom used for action and with weekly epidemiologic bulletin at National level having been discontinued in the past one year means that surveillance information is not widely disseminated. PHEM focal points not aware that AEFI are under surveillance revealing the obvious gaps of information exchange between the EPI and PHEM in effect AEFI surveillance is not done. AEFI guidelines and reporting forms were not available in many of the health facilities across regions visited and the awareness on the reporting protocol lacking at Woreda and Health facilities levels. In 2015 regular meetings of NPEC have not been held. Persistent stringent customs clearance regulations continue to persist thereby causing frequent stock outs and loss of perishable materials. Moreover, NPENT isolation rate has remained below the targeted 10%. The implementation of the equipment maintenance plan is still outstanding. After many years of operation, the Regional laboratory data is not yet merged with National laboratory data and presently there is limited laboratory capacity to support investigation and testing of Yellow fever samples and other dangerous pathogens.

With respect to *Somalia* the weaknesses include the existence of copies of old guidelines printed in 2006 and used in field and at regional level. Collecting 3 contact samples from each notified AFP case is a standard strategy but was not adhered to in Somalia. Furthermore, issues like “Hot cases” and clustering are not implemented systematically. There was no clear strategy for surveillance or mapping of special populations like the nomads. Excluding AFP cases was noticed as a common practice in Banadir with no clear base or standard measures. Excluding is done by DPOs and PEOs with no documentation, validation or expert review. Reason given for excluding the AFP case was that paralysis was not flaccid.

Banadir Hospital and high priority hospital in Sahil and Hargeisa were not identified as a high priority to be visited by a qualified staff who can deal with physicians without being intimidated. A newly established Neurology Hospital in Hargeisa was also missed as it was not included on list of active sites. Monitoring trends of reporting is done at central level and is not used to prioritize facilities for active surveillance. The focal person for surveillance in Gabiley, a major Hospital, in Hargeisa displayed complete ignorance of the surveillance process predisposing to missing AFP cases. The same Hospital would not allow the District Polio Officer (DPO) to access the Hospital premises. There was limited awareness and contribution from staff examining above 5 years children in most health facilities visited.

No documented plan or monitoring and supervision of active surveillance and no documented evidence of any supervision conducted. No standard supervisory tool. Locally developed supervisory check lists as well as supervisory logbooks were seen in limited places. Forms for laboratory results were sometimes missing and results were reported verbally with no record in the files. Data validation at lower levels and with the AFP case is not a routine process in insecure areas hence cases are mostly validated by PEOs and DPOs who are less qualified to conduct such examinations. Though “Hot AFP cases” were flagged at lower levels, there was no evidence of follow up to prioritize laboratory investigation at the national level. Sixty days follow up is not carried out by physician in all cases. Detailed investigation of AFP cases with zero doses OPV is not a routine. Increase in AFP cases detected was not noticed despite the on-going outbreak despite claims that AFP surveillance had been enhanced. The centrally documented bulletins do not reach the peripheral level. Capacities for data analysis were lacking at zonal and regional level leading to limited analysis of performance indicators at those levels. Though the ZERO reporting forms are available at various levels, analysis and monitoring of completeness/timeliness of zero reporting not taking place at operational level.

Stool collection kits, cold boxes and ice packs are not always available at district because DPOs do not have offices. In some cases, kits are kept in regional office which might cause delays in specimen collection. Marked delays in stool specimens reaching the laboratory. 0nly 3% of stool specimens reached the lab in Nairobi within the recommended 3 days due to bad roads from health Facilities and lack of transport means. Delays from regional level to the lab in Nairobi are due to the flight schedules. The issue of ownership cannot yet be realized due to the unavailability of resources to support the activities as the country is still in conflict in most areas. The system is therefore dependent on Donor support. This arrangement is quite expensive and my not be sustainable. Arising from the fact that verification at the sources of data collection is compromised by the bad security situation, the quality of data is quite inadequate. Several variables are not fully completed in the case investigation forms. No Certification Committees have been set up in the country yet because of the nonavailability of qualified personnel as well as the support mechanisms to allow these committees to function.

## Discussion

This AFP surveillance review for the Horn of Africa was able to identify gaps at the various levels within the context of a health system, which were most commonly related to the challenges of funding, training, and supervision^8^. Shortcomings with surveillance system were particularly evident at the local level. Although AFP surveillance met national performance standards in some of the Horn of Africa countries reviewed, widespread deficiencies and the limited resources were observed and reported at subnational levels^9,10^.

In Kenya the surveillance structure and network were in place from the national to the community level. However, the performance of the system in Kenya was not robust enough to allow timely detection of transmission of WPV/cVDPV The surveillance review noted crucial gaps in areas of funding. Funds were allocated for surveillance at the National level, most Counties and Sub-Counties, but Surveillance funds provided through WHO are not reaching operation levels promptly. AFP surveillance indicators were below required operational standard in a third of the sub-counties. There was also gross inadequacy in data quality management and inability to provide feedback to guide actions at all levels. Active surveillance not performed according to the national guidelines. Knowledge gap on VPD surveillance were very evident at health facility level. Inadequate implementation of the 2012 surveillance review recommendation.

The situation in Somalia was very much similar to that in Kenya and sometimes more disturbing. AFP surveillance system in Somali land and Puntland was well developed and sensitive enough to timely detect polioviruses. There does not appear to be any undetected circulation of polioviruses in both zones. However, in south and central zones there was high level of insecurity which did not allow for adequate surveillance. Exclusion of notified AFP cases reported by the community has been a practice in Central zone/ Banadir with no documentation or expert reviewing. Banadir might have missed wild or VDPV importation or circulation.

The situation was the same in Ethiopia. Overall, a good surveillance system was in place in Ethiopia with involvement of Government and private health facilities as well as community based structure. However, sensitivity of surveillance needed to be strengthened further to detect all transmission timely. There has been significant improvement in core surveillance indicators in 2015. There is strong system of surveillance at all levels with PHEM taking lead. Reporting network is extensive however, some of the important private practitioners/ traditional healers are still not involved systematically. System of prioritization of reporting sites for active surveillance visits exists. However, frequency and quality of visits is suboptimal. Systems of training of surveillance focal persons existed but there were gaps in knowledge at different levels and high turnover a challenge. Review of surveillance, data analysis and use for action including review meetings at different level was not systematic. Persistent issues related to customs clearance of important reagents/ supplies. NPENT isolation rate has remained below the targeted 10%. Weekly reporting system exists at all levels; hard copies are not being transmitted to the next level.

These gaps were further confirmed by the prolonged and serial outbreaks and sustained transmission of wild poliovirus post importation in the Horn of Africa. The gaps identified in Kenya’s surveillance, for instance, forewarned partners of the risk of an impending regional wild poliovirus outbreak in these high-risk districts, which subsequently occurred in North Eastern province^8^. Detection of surveillance gaps within the national program led to planning for systemic modifications and trainings to improve the suboptimal areas identified by the evaluation.

The review led to recommendations that helped the national programmes to strengthen surveillance through trainings and prioritizing of hard-to-reach and migratory populations, as well as use of short message service (SMS) and other reporting technologies, active surveillance at the community level, environmental surveillance, and strengthening partnerships with other health programmes. It also led to the identification of the numerous constraints faced in the south and central zones of Somalia and the formation and use of village polio volunteers, which helped in the slowing down the transmission of poliovirus in the Horn of Africa.

Consequently, the review came up with a number of recommendations to strengthen the AFP surveillance systems in the Horn of African countries. The review recommended that ministries of health at various levels in collaboration with partners should improve data quality management and feedback at all levels. This will require building the capacity of key staff in the use of DHIS-2 to analyze data on priority diseases for decision making. The practice of weekly data harmonization among ministries of health, polio laboratories and WHO country offices were encouraged and the ministries of health at all levels were also encouraged to update contact lists and share weekly IDSR bulletin with lower levels.

It was also recommended that the lower levels of the ministry of health should ensure that the national surveillance unit receives a copy of the case investigation forms both for AFP and other vaccine preventable diseases as soon as the samples reach the national levels to take immediate action where necessary. The ministry of health and local authorities in collaboration with partners should strengthen the community participation in the surveillance of vaccine preventable diseases. Deliberate steps should also be taken to improve the communication between the health facility surveillance focal point and the community volunteers especially in insecure areas.
